# Fine needle aspiration as a diagnostic tool in cysticercosis: a case series

**DOI:** 10.1186/s13256-018-1957-8

**Published:** 2019-03-29

**Authors:** Tummidi Santosh, Nagiredla Puneeta, Manoj Kumar Patro, Pravin Gaikwad

**Affiliations:** 1 Department of Pathology & Laboratory Medicine, AIIMS, Bhopal, MP India; 2 Department of Oral and Maxillofacial Pathology, Maitri College of Dentistry and Research Centre, Anjora, Durg, CG India; 3 Department of Pathology, Government Medical College & Hospital, Balangir, Odisha India

**Keywords:** Rapid on-site evaluation, Toluidine blue, Fine needle aspiration cytology, Cysticercosis

## Abstract

**Background:**

Cysticercosis is a zoonotic disease. It is caused by the larval form of the pork tapeworm *Taenia solium*. This disease is a public health problem in a country like India, but its incidence is underestimated. With the advent of fine needle aspiration cytology with rapid on-site evaluation, early detection of this disease is possible, especially when the lesion is in anatomically approachable superficial locations.

**Case report:**

We had four cases of cysticercosis diagnosed by fine needle aspiration cytology with on-site evaluation using toluidine blue stain, followed by Giemsa and Papanicolaou stain. Our cases included three Hindu male patients of age 30 years, 23 years, 17 years and an 26 year old Hindu female.

**Conclusion:**

Fine needle aspiration cytology diagnosis of cysticercosis can be easily made provided the reporting cytologist is aware of the morphological criteria. Rapid on-site evaluation can further help in taking additional material and caution during staining process.

## Background

Fine needle aspiration cytology (FNAC) is a well-recognized diagnostic procedure for the evaluation of inflammatory nodules caused by parasites. The diagnostic role of FNAC in cysticercosis was first emphasized by Kung *et al.* in 1989 [1]. Since then, a spectrum of cytological details of cysticercosis covering the entire range, from viable cysts through to necrotic and calcified lesions, has been described [2]. The possibility of cysticercosis should be kept in mind during assessment of all inflammatory and cystic swellings. We report four cases of cysticercosis diagnosed by rapid on-site evaluation (ROSE) using toluidine blue; we emphasize some simple interpretive aspects using ROSE and its practical value, especially for cytopathologists with limited exposure.

## Case presentation

### Case 1

**Fig. 1:**
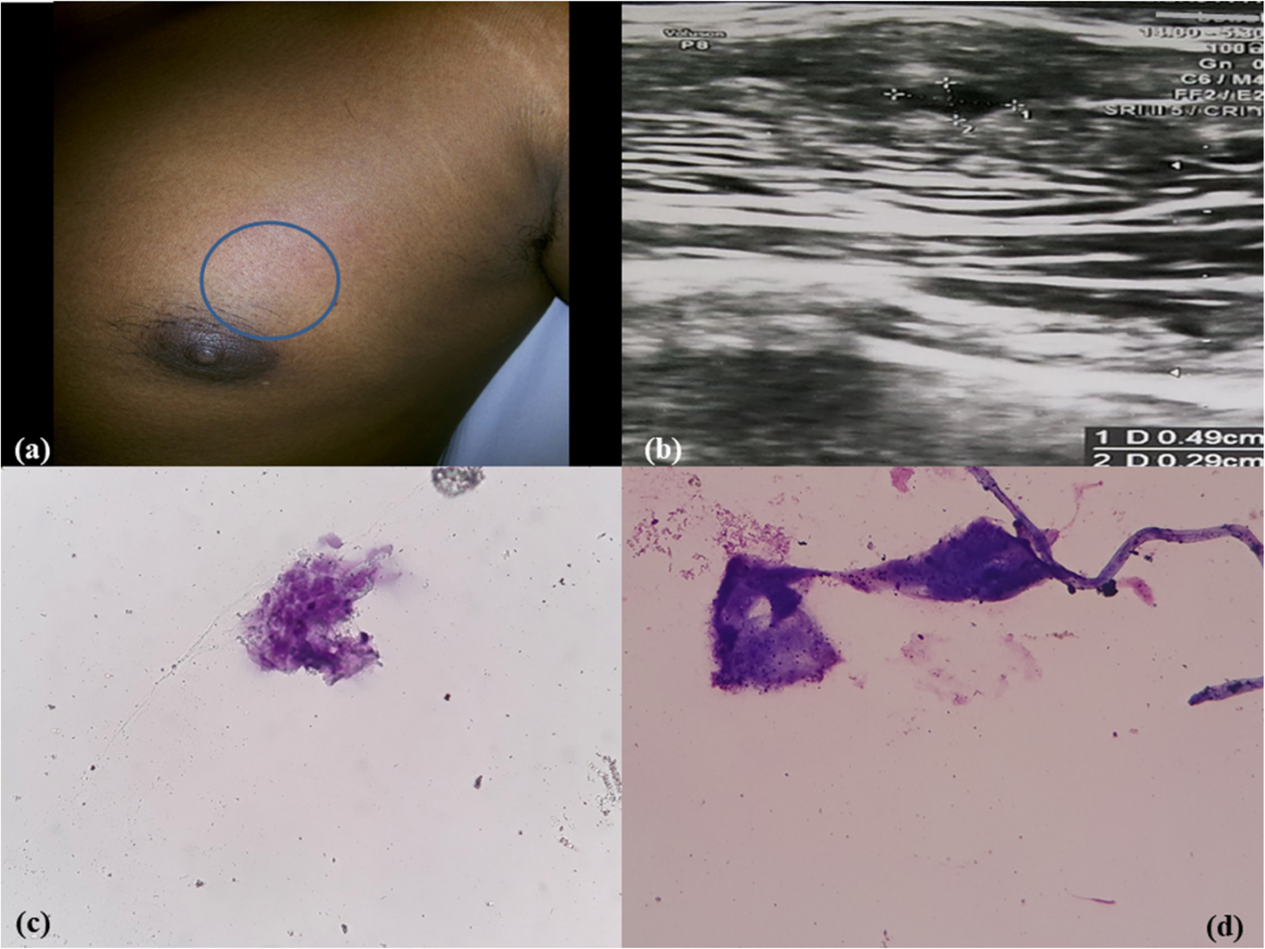
Case 1. **a**, **b** Swelling in left chest wall. Ultrasonography showed a small cystic lesion of 0.5 × 05 mm in left chest wall. **c**, **d** Cytology revealed a fragment of parasite intestine and degenerated remnants of parasite with inflammatory background. (Toluidine blue, × 10)

A 30-year-old Hindu man, vegetarian by diet, presented with complaints of chest pain and swelling for 4–5 months. He had a history of a swelling that intermittently increased in size and restricted shoulder movement. He was a poultry worker by profession. His socio-economic status was poor. Ultrasonography (USG) of his chest wall showed a small cystic lesion of 0.5 × 0.5 mm in the left-side of his chest wall with adjacent heterogeneous muscle; this indicated a possibility of left-sided chest wall intramuscular cysticercosis or an old hematoma. FNAC yielded 1 ml of granular, whitish fluid-like material (Fig. 1a, b).

### Case 2

**Fig. 2:**
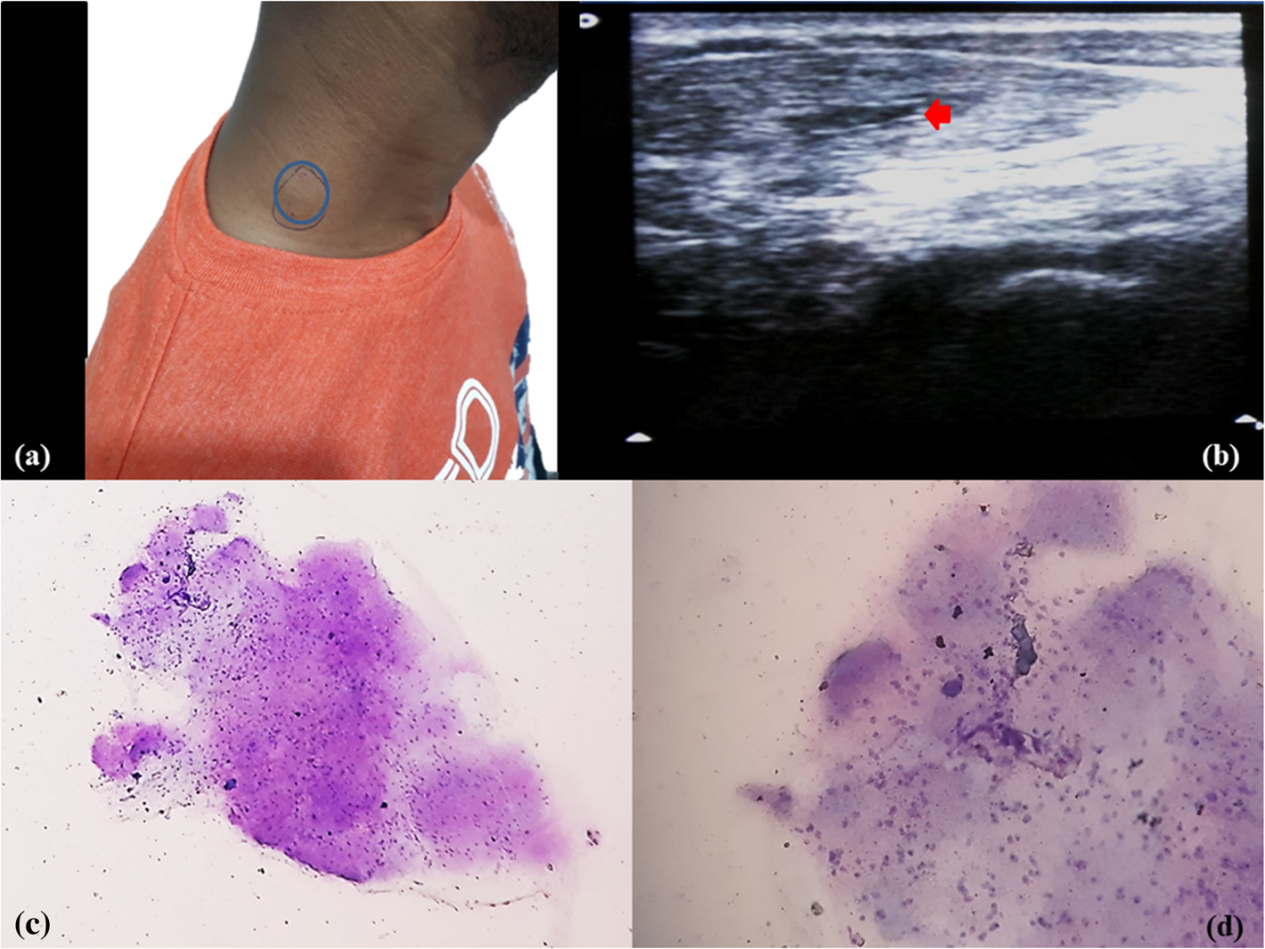
Case 2. **a**, **b** Swelling in right neck region; ultrasonography of the neck showed a 2 × 1.5 cm irregular cystic lesion in right scalene muscle. **c**, **d** Cytology showed granular bladder wall with subcuticular cells along with small pyknotic-looking nuclei and fibrillary parenchyma and inflammatory cells. (Toluidine blue, ×10 & x40)

A 23-year-old Hindu man, non-vegetarian by diet, presented with right-side neck swelling for 1 month. He had a history of right-side ear ache. USG of his neck showed a 2 × 1.5 cm irregular cystic lesion in right scalene muscle, possibly cysticercosis. FNAC was done which yielded 0.6 ml of whitish fluid-like material. Post FNAC, he had a reddish allergic reaction at local site (Fig. 2a, b).

### Case 3

**Fig. 3:**
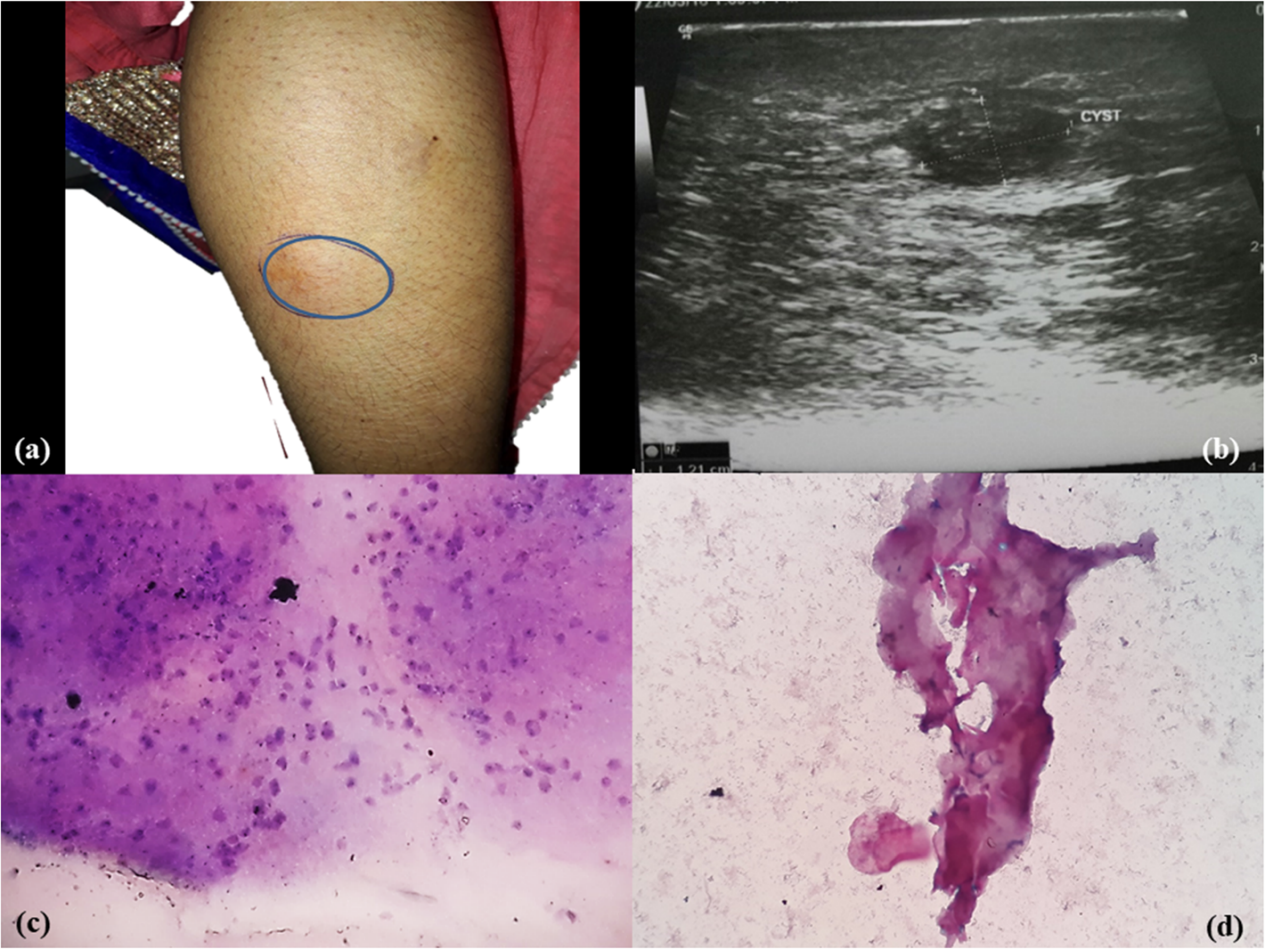
Case 3. **a**, **b** Swelling in right calf lateral aspect; ultrasonography showed a right lower limb small hypoechoic lesion in intramuscular plane measuring 1.2 × 0.7 cm. **c**, **d** Cytology showed granular bladder wall with subcuticular cells, small pyknotic-looking nuclei, fibrillary parenchyma along with inflammatory cells and homogenous acellular material. (Toluidine blue, × 40)

A 26-year-old Hindu woman, pure vegetarian in diet, presented with complaints of right lateral calf swelling in the past 7 to 8 months. She had a history of chronic intake of salad and uncooked green vegetables. She had a history of intermittent increase in size of the swelling with radiating pain to lower extremity. The swelling subsided on hot bathing. No other swelling was noted and there was no history of trauma. USG of her right lower limb revealed a small hypoechoic lesion in intramuscular plane measuring 1.2 × 0.7 cm. On examination, the swelling over her right lateral calf region was an intramuscular, soft to firm cystic lesion that was 1.5 × 1 cm in size (Fig. 3a, b). The overlying skin was reddish with itching and tenderness. Aspirate of fine needle aspiration (FNA) was 0.5 ml of whitish granular material.

### Case 4

**Fig. 4:**
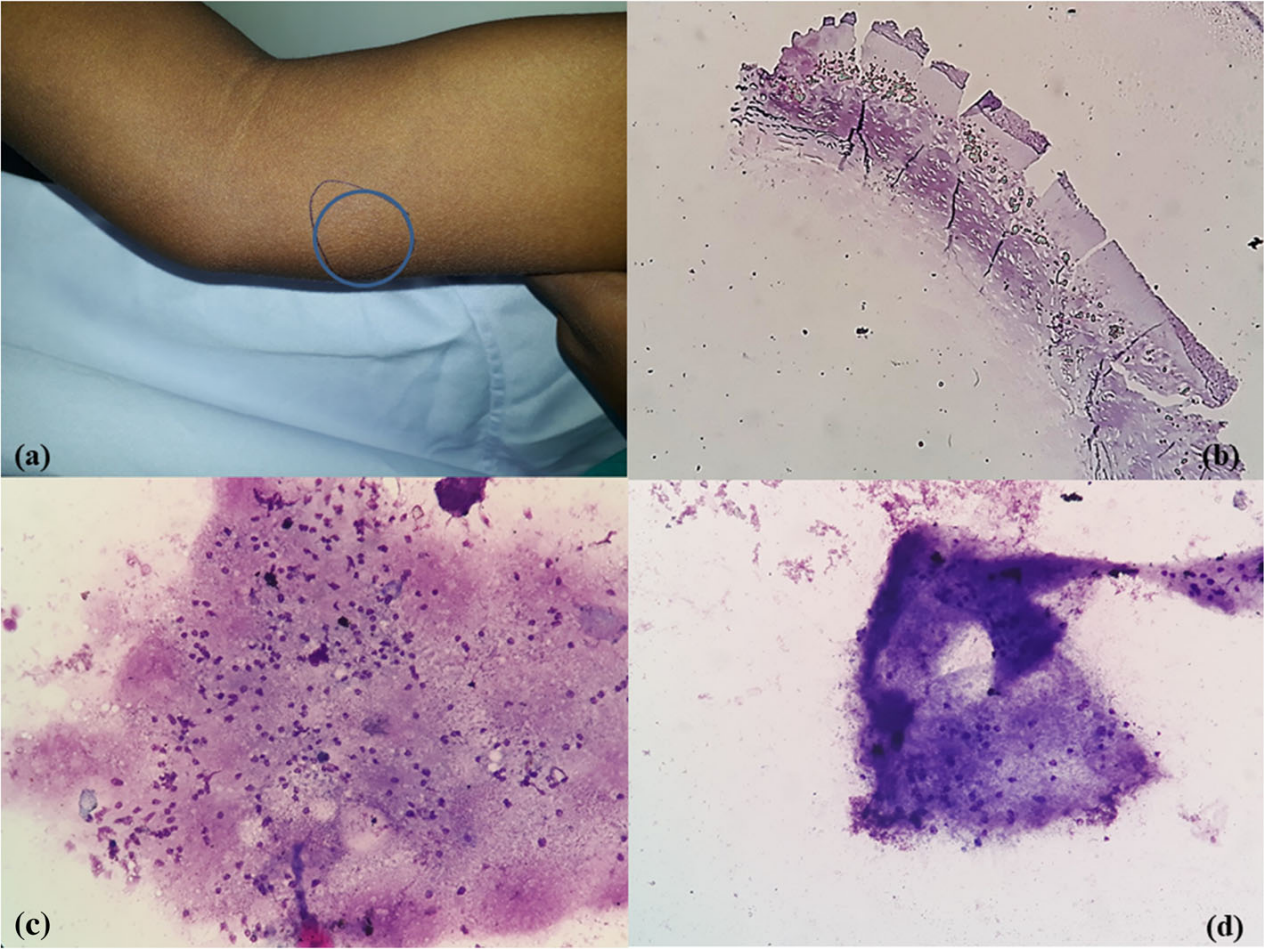
Case 4. **a**, **b**, **c**, **d**: Swelling in right medial aspect of arm. Cytology showed the intestinal wall of cysticercosis, calcified debris, and granular bladder wall with subcuticular cells with small pyknotic-looking nuclei. (Toluidine blue, x10, ×40)

A 17-year-old Hindu boy, a vegetarian by diet, presented with swelling in the medial aspect of his right arm for 1 year. The swelling had gradually increased in size during the past 1 month. He had a history of itching near the swelling. On examination the swelling measured 3 × 2 cm; it was well defined and soft, and an itching scar mark was noted (Fig. 4a, b). No other swelling was seen. There was no history of trauma. A routine complete blood count was normal except for mild anemia. FNA yielded a drop of straw-yellow granular fluid.

### Cytological findings

FNAC of all the four cases revealed a very similar picture. Smears were paucicellular showing polymorphs, histiocytes, and occasional lymphoid cells. Many histiocytic aggregates were seen along with a few histiocytes with ingested debris. Also seen was a portion of cysticercosis, bladder wall, and many small non-human parasite nuclei and surrounding inflammation. Two of the cases revealed background calcified debris and hooklets. Scattered at places were the walls of cysts of cysticercosis (Figs. 1c, d; 2c, d; 3c, d; 4b, c, d). The cases were then reported as cysticercosis.

## Discussion

Cysticercosis is an infection of both humans and pigs with the larval stages of the parasitic cestode, *Taenia solium*. This infection is caused by ingestion of eggs shed in the feces of a human tapeworm carrier. Pigs and humans become infected by ingesting eggs or gravid proglottids. Humans are infected either by ingestion of food contaminated with feces, or by autoinfection. In the latter case, a human infected with adult *T. solium* can ingest eggs produced by that tapeworm, either through fecal contamination or, possibly, from proglottids carried into the stomach by reverse peristalsis. Once eggs are ingested, oncospheres hatch in the intestine, invade the intestinal wall, and migrate to striated muscles, as well as the brain, liver, and other tissues, where they develop into cysticerci [3].

Fully developed cysticerci are opalescent, milky white cysts, elongated to oval, and approximately 1 cm in diameter. The cyst contains fluid and a single invaginated scolex. The scolex has a rostellum, four suckers, and 22–32 small hooklets. The cyst wall is multilayered, 100–200 mm thick, and covered by microvilli. The outer, cuticular layer appears smooth and hyalinized and is frequently raised in projections [4]. Beneath the tegument is a row of tegumental cells. The inner layer or parenchyma is loose and reticular, containing mesenchymal cells and calcareous corpuscles [5]. The calcareous corpuscles are a unique feature of cestode tissue. These spherical, noncellular masses occur in the parenchyma and are especially prominent in larval cestodes. The corpuscles take on a bluish-purple color in hematoxylin and eosin (H and E) [6].

The cysticercus secretes certain substances locally (for example, paramyosin, taeniastatin), which alter the host immune response. Both cellular as well as humoral immunity are affected [7, 8]. With passage of time, these mechanisms become ineffective, and the inflammatory response leads to degeneration of the parasite, granuloma formation, and calcification.

The prevalence of cysticercosis ranges between 7 and 26%. The clinical manifestations depend on the location and number of lesions at a site [8]. The most frequent sites affected are skeletal muscles, subcutaneous tissue, brain, ocular tissue, heart, liver, lungs, and peritoneum [9]. A majority of cases do not lead to clinical ill-health, except occasional abdominal discomfort, anorexia, and chronic indigestion. Straying of proglottids may sporadically cause appendicitis or cholangitis. The most serious risk of *T. solium* infection is cysticercosis [10].

FNAC in combination with ROSE has been shown to increase diagnostic yield and accuracy. Numerous studies have documented an increase in adequate specimens after the implementation of ROSE compared to FNA performed before ROSE was available. The decrease in non-diagnostic specimens has been reported to drop from 15–47% to 4–23%, depending on the FNA site [11, 12]. The improved adequacy rate is largely due to additional passes performed at the time of FNA, when the initial pass is non-diagnostic. This procedure ensures that sufficient quantities of cells of adequate quality are obtained to permit a complete diagnostic workup. Ultimately, this will translate into an appropriate treatment plan without the patient undergoing any invasive surgical procedures [13].

Aspiration of clear fluid is a strong indicator of a parasitic infection in a palpable subcutaneous or intramuscular nodule, which provides a clue for the diagnosis of cysticercosis [5]. Pooja and Pratima had 69.3% cases where the aspirate was clear fluid, varying in quantity, whereas aspirate was purulent in 16.8% cases, blood mixed in 13.1%, and granular or particulate in 0.8% cases [8].

The cytological diagnosis is quite straightforward in cases where actual parasite structure is identified in the smears. It initially comprises macrophages and lymphocytes followed by the appearance of palisaded histiocytes. Eosinophils and plasma cells appear still later. Subsequently, neutrophils surround and invade the parasite and lead to its degeneration. However, in other cases, the presence of histiocytes, which may be in palisaded clusters or not, a typical granular dirty background, and so on, are the features which should always alert a pathologist to this possibility. Epithelioid cell granulomas can also be present in the later stages. Foreign body giant cells are invariably present in surrounding inflammatory zone. However, in some cases of cysticercosis, none of these features may be present, and the inflammatory infiltrate may also be variable. Demonstration of fragment of larval bladder wall, hooklets, and calcareous corpuscles confirms the diagnosis of cysticercosis [2, 4].

Control measures include: proper cleaning and cooking of vegetables, meat inspection, health education, and adequate sewage treatment and disposal. The drug of choice is albendazole 10–15 mg/kg body weight per day given twice daily with a fatty meal. Seven to 14 days may be sufficient for some patients, but a longer course (up to 28 days) is advisable at present. It can be repeated as necessary. It can even be combined with a steroid for control of inflammation [3, 10].

## Conclusion

FNAC with ROSE helps in the early diagnosis of cysticercosis along with helping to take additional material for serological studies if required. We should note that cysticercosis is more common in our part of the world, than usually thought. A cytopathologist needs to have in mind that all cystic/inflammatory lesions have the possibility of the condition.
